# Individual plant genetics reveal the control of local adaptation in European maize landraces

**DOI:** 10.1186/s12915-025-02241-8

**Published:** 2025-05-21

**Authors:** Leke Victor Aiyesa, Dietrich Kaufmann, Birgit Zumbach, Wolfgang Link, Stefan Scholten, Timothy Beissinger

**Affiliations:** 1 Division of Plant Breeding Methodology, Department of Crop Sciences, Faculty of Agriculture, Georg-August-University of Göttingen, Göttingen, Germany; 2 Division of Crop Plants Genetics, Department of Crop Sciences, Faculty of Agriculture, Georg-August-University of Göttingen, Göttingen, Germany; 3 Heritable Agriculture, Mountain View, CA 94040 USA; 4 Centre for Breeding Research (CiBreed), Georg-August-University of Göttingen, Göttingen, Germany

**Keywords:** Individual plants, Nucleotide diversity, Founder event, Selection signatures

## Abstract

**Background:**

European maize landraces encompass a large amount of genetic diversity, allowing them to be well-adapted to their local environments. This diversity can be exploited to improve the fitness of elite material in the face of a changing climate.

**Results:**

We characterized the genetic diversity of 333 individual plants from 40 European maize landrace populations (EMLPs). We identified five genetic groups that mirrored the proximities of their geographical origins. Fixation indices showed moderate differentiation among genetic groups (0.034 to 0.093). More than half of the genetic variance was observed to be partitioned among individuals. Nucleotide diversity of EMLPs decreased significantly as latitude increased (from 0.16 to 0.04), suggesting serial founder events during maize expansion in Europe. GWAS with latitude, longitude, and elevation as response variables identified 28, 347, and 68 significant SNP positions, respectively. We pinpointed significant SNPs near *dwarf8*,* tb1*,* ZCN7*,* ZCN8*, and *ZmMADS69* and identified 126 candidate genes with ontology terms indicative of local adaptation in maize, regulating adaptation to diverse abiotic and biotic environmental stresses.

**Conclusions:**

This study suggests a quick and cost-efficient approach to identifying genes involved in local adaptation without requiring field data. The EMLPs used in this study have been assembled to serve as a continuing resource of genetic diversity for further research aimed at improving agronomically relevant adaptation traits.

**Supplementary Information:**

The online version contains supplementary material available at 10.1186/s12915-025-02241-8.

## Background

The domestication of maize (*Zea Mays L.*) occurred around 9000 years ago in the Balsas River Valley of southern Mexico. Maize was introduced to Europe in 1493 through the Caribbean by Christopher Columbus during his second voyage [1]. The cultivation of maize in northern European regions was first reported in Germany in 1539, followed by a rapid expansion that led to immense diversification and adaptation to long days and low temperatures [2]. There are also claims of secondary introductions to different northern parts of Europe from North America [2, 3]. Currently, maize landrace populations are cultivated globally across a vast geographic range, from 58°N latitude in northern Europe, spanning temperate, subtropical, and tropical regions, to 40°S latitude. Additionally, maize is grown at altitudes as high as 3000 masl in regions like the Andes and the Himalayas [4–6]. This extraordinary adaptability underscores maize’s importance as a model for studying plant domestication and local adaptation [7].

Previous reports have characterized European maize landrace populations (EMLPs) within and across countries utilizing morphological differences, such as the number of days to flowering [8–10]. EMLPs collected from northeastern Europe for instance were noted for earlier flowering compared to those from southern Europe [9, 10]. However, these characterizations are often affected by environmental fluctuations, which can obscure underlying genetic identity [9]. The extensive adoption of molecular markers, such as isozyme, restriction fragment length polymorphisms (RFLP), and single sequence repeats (SSRs) [11–13], and more recently, single-nucleotide polymorphisms (SNPs) from SNP arrays [14–18], largely mitigated this issue. However, discovering large and novel polymorphisms, especially through genotyping-by-sequencing (GBS), should provide a broad genetic base and prevent misinterpretation of diversity studies due to ascertainment bias [19, 20].

The rich genetic and geographic diversity in maize landraces holds significant potential for enhancing the adaptability of elite maize lines to changing climatic conditions [5, 21]. However, the selection of a limited number of parental landraces to develop the elite lines currently used in Europe has not fully captured this diversity [22]. As a result, some favorable alleles have been lost, limiting the potential for these lines to effectively adapt to extreme climatic conditions [23]. Furthermore, as climate change progresses, previously neutral or even negative alleles may become beneficial for local adaptation, altering the value of populations that were once considered less important [23]. Local adaptation occurs when a population exhibits higher fitness traits than non-local populations [24]. In maize landraces, this phenomenon is complex, and its understanding remains incomplete [24, 25]. To study local adaptation, researchers have used methods such as multi-environment trials [5], reciprocal transplantation [24, 26, 27], and common garden experiments [28–30] to observe phenotypic variation and identify functional variants related to adaptation. While these methods have provided valuable insights, they are labor-intensive, costly, and time-consuming. By leveraging the geographic information of maize landraces and its impact on genetic diversity, valuable insights can be gained on the genetic basis of their local adaptation, particularly for altitudinal variations [2, 5], as well as variations in longitude and latitude [31–34].

In this study, we carried out extensive genetic screening of EMLPs using whole genome shotgun sequencing of individual plants to understand their population structures and investigated patterns of genetic diversity across the geographic origin. We further identified genomic loci under selection for adaptation to local environments, leveraging each population’s geographical information.

## Results

### SNP dataset

**Fig. 1:**
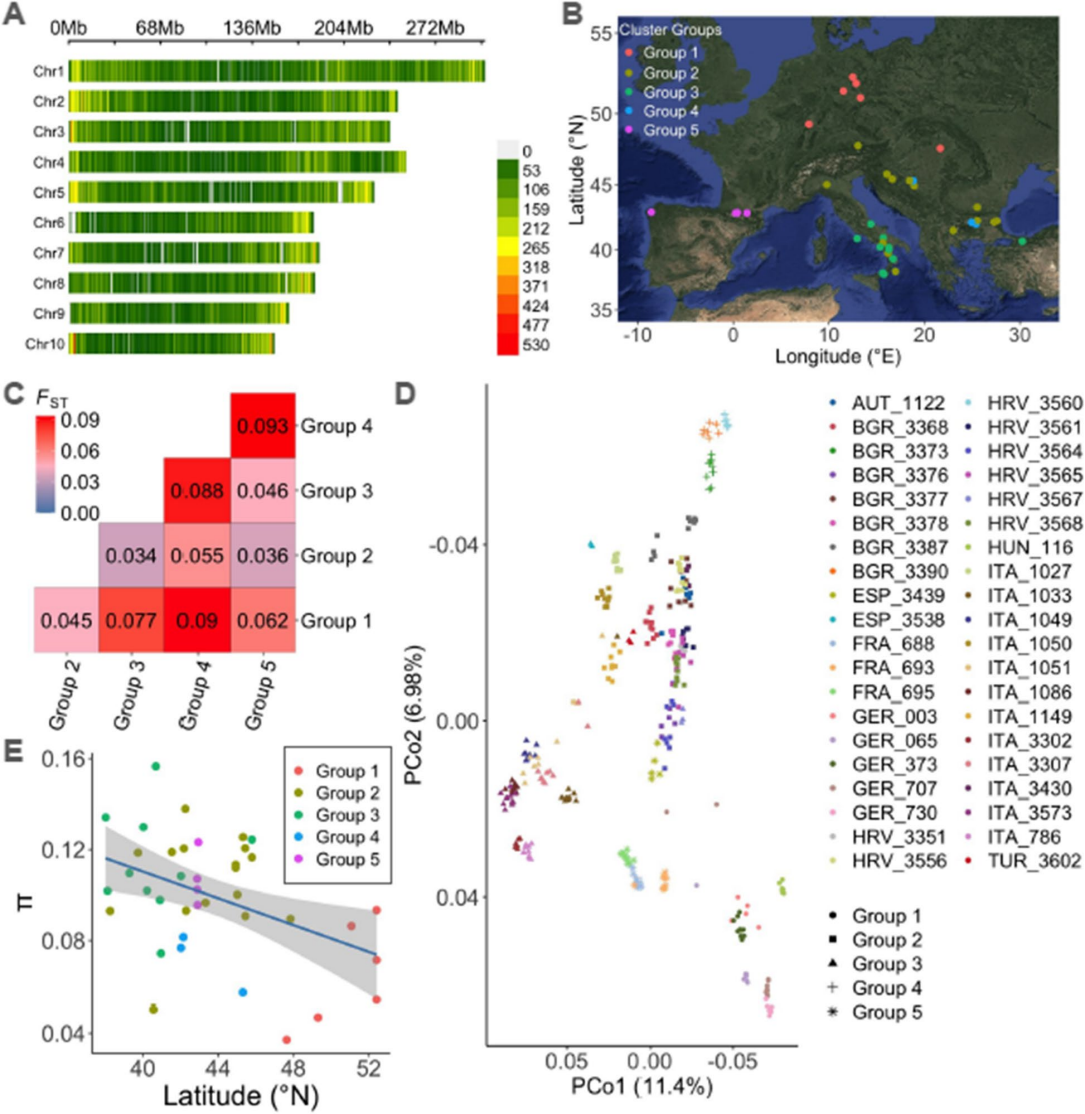
Distribution of SNPs within and across chromosomes showing SNP density per 1 Mb (**A**). Geographic distribution of the 40 European maize landrace populations colored by the optimal number of genetic groupings at *K* = 5 (**B**). Genetic differentiation using Fixation indices (F_ST_) among the genetic groups (**C**). Principal coordinate analysis (PCoA) showing the clustering of 333 individual plants, colored and shaped according to the population and genetic groups to which they belong (**D**). Regression of latitude on nucleotide diversity (π) of the 40 populations revealing a serial founding event (**E**)

We conducted genotyping-by-sequencing (GBS) on 340 individual plants from 40 landrace populations originating from nine countries, representing a broad geographical distribution of maize across Europe (Additional file 1: Fig. S1). After final variant filtering, we retrieved 152,671 SNPs from 333 individual plants (Fig. 1A). These SNPs were distributed across the 10 chromosomes, ranging from 10,677 to 23,543 SNPs for chromosomes ten and one, respectively. SNPs were well distributed within chromosomes, except for centromeric regions, which showed a lower SNP density. This result is consistent with reported GBS SNP data for maize populations and the relatively low SNP density at centromeres and peri-centromeres is expected due to low recombination [35, 36]. The distribution of genome-wide minor allele frequencies (MAF) and heterozygosity were as expected (Additional file 1: Fig. S2).

### Genetic clustering and genetic differentiation of EMLPs

Analyzing diverse landrace populations across a wide range of geographical origins helps to capture the broad genetic diversity available in maize [37, 38]. To confirm this, we examined the relationship between the geographic distance (using latitude, longitude, and elevation of origin) and genetic distance (determined by SNP markers) of EMLPs. The Mantel test [39] revealed a significant correlation of 0.41 (*p*-value < 0.001) between pairwise geographic and genetic distances. We further identified genetic groups based on hierarchical clustering analysis at *k* = 5 (Additional file 1: Fig. S3). These groups closely mirrored the geographical proximities of EMLPs, as presented in the principal coordinate analysis (Fig. 1B, D). This provides a broader view of reported classifications of EMLPs into northeastern populations (group 1), southeastern populations (group 2 and group 4), south-Italian populations (group 3), and the Pyrenees populations (group 5) [2, 30–39]. Group 2 exhibited the largest spread of populations across three southeastern countries and southern Italy, possibly due to the hybridization of tropical dents and northern flint populations that occurred in this region, reported to be the European corn belt [2, 30].

We further assessed genetic differentiation between the five genetic groups using their pairwise fixation indices (*F*_*ST*_) [40]. *F*_*ST*_ ranged from 0.034 between Group 2 and Group 3 to 0.093 between Group 4 and Group 5 (Fig. 1C). We observed a high *F*_*ST*_ between the northern and southern groups, consistent with previous reports for European maize landraces [41] and between the Pyrenees (west) and southeastern groups. Interestingly, for three out of the nine countries of origin (Bulgaria, Croatia, and Italy), we found populations belonging to different groups, contrary to our expectations. The mixtures of group 2 and group 3 found among south Italian populations could be attributed to differences in elevation. While group 2 south Italian populations were found in the lowlands with an average elevation of 245.6 masl, group 3 populations belonged to relatively higher elevations with an average elevation of 690 masl (Additional file 1: Fig. S4).

### Nucleotide diversity of EMLPs and founder effects

Genetic variation between maize populations was low compared to within populations as observed in the analysis of molecular variance (AMOVA) which partitioned 51% of the genetic variance to individuals within populations, consistent with reported estimates [42, 43], 36.4% between populations, and 12.6% between groups (Additional file 1: Fig. S5). The genotyped individual plants for each population were used to estimate the population’s nucleotide diversity (π), using the average of pairwise differences between all possible pairs of individuals in the population. A high π value would indicate that such a population consists of more genetically distant individuals, and a low π indicates that such a population consists of less genetically distant individuals (Additional file 1: Fig. S6). The mean values of π for the populations ranged from 0.04 for a Hungarian population (HUN_116) to 0.16 for a southeastern population (TUR_3602), while the average π value was 0.106 across populations (Additional file 1: Fig. S6). Our values are comparable to reported π for maize landraces [10, 33, 44, 45]. Interestingly, we observed a significant decline (*p*-value = 0.005) in π as latitude (°N) increased (Fig. 1E), which suggests a serial founder event resulting in the northeastern populations. This aligns with reports on maize introduction into Europe and utilization that led to major expansion due to selection of EMLPs from the subtropical south to the temperate north and northeastern regions [2, 9, 30, 34].

### GWAS identifies genomic regions under selection

**Fig. 2:**
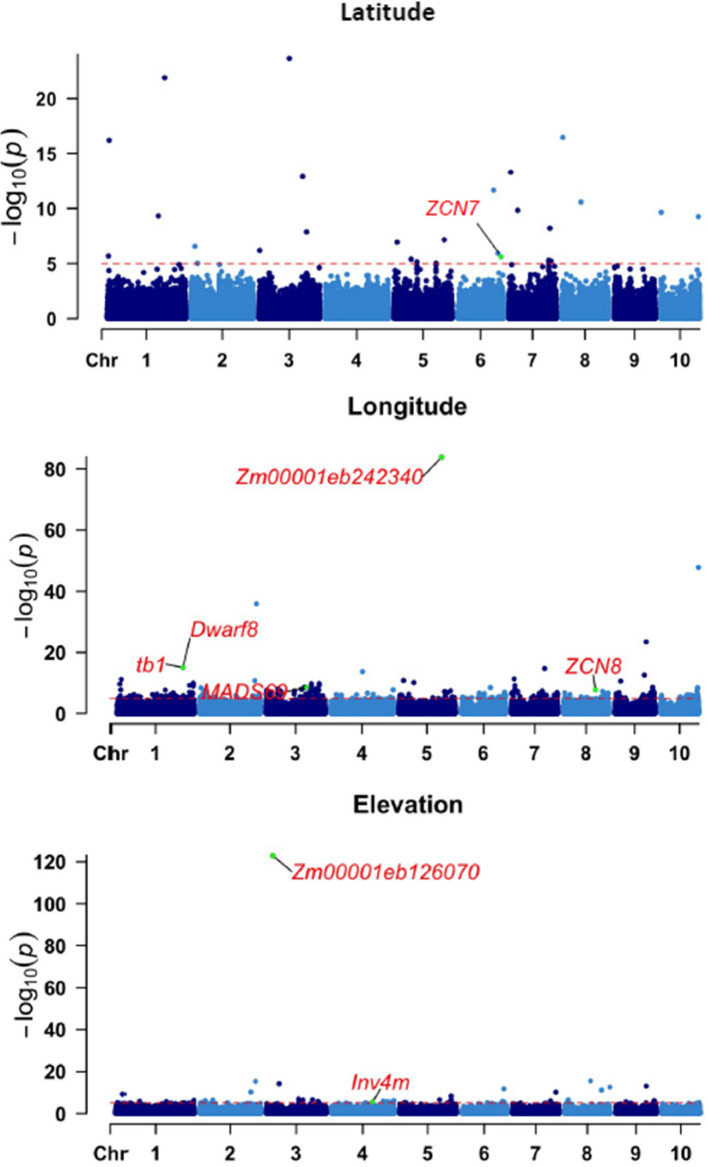
GWAS results for latitude, longitude, and elevation. The *y*-axis depicts the negative log₁₀-transformed *p*-values, while the *x*-axis represents the positions of SNPs across the maize chromosomes (labeled as'chr'). The red horizontal line at –log₁₀(5) (corresponding to a *p*-value of 0.00001) indicates the significance threshold for SNP associations. Some of the notable genes reported for plant height and flowering time (highlighted in red), co-localize with significantly associated SNP positions identified in this study

Maize landraces are genetically diverse materials that have since long adapted to their local environments, enabling researchers to explore their geographical properties in identifying genetic controls for local adaptation [5]. Prompted by this finding, we conducted GWAS using the latitude, longitude, and elevation of origin of our EMLPs as response variables and the 152,671 SNPs as explanatory variables. We corrected the population structure using the first three principal coordinates, explaining 22.8% of the total variation and a Fixed and Random Model Circulating Probability Unification (FarmCPU) algorithm [46]. We identified 28, 347, and 68 significant SNPs, for latitude, longitude, and elevation respectively, flagged at *p* < 0.00001 (− log10^5^) to allow less than three false positives (Fig. 2). We found highly significant association with *p*-values 1.47e − 123 for elevation on chromosome 3, position 26.4 Mb, 1.32e − 84 for longitude on chromosome 5 at 171.3 Mb, and 2.23e − 24 for latitude on chromosome 3, at 119 Mb. We compared some of the significant SNP positions with reported genomic positions for flowering time and plant height, which are indicator traits of adaptation in maize [24, 47–49], and found overlapping peaks. Notably, on chromosome 4, we identified two significant associations for elevation between 150 and 200 Mb. This chromosome region has been reported for *In4vm*—an introgression from highland Mexicana to highland maize consisting of floral genes [5, 7]. We also found five significant SNPs located in the vicinity of *dwarf8* (221–225 Mb) for latitude, and two significant SNPs close to *tb1* (261 Mb and 267 Mb) on chromosome 1 for longitude. *ZCN7* (ZEA CENTRORADIALIS7, on Chromosome 6, 171.3 Mb) and *ZCN8* (ZEA CENTRORADIALIS8 on Chromosome 8, 128.5 Mb) were identified for longitude. These genes have been reported to regulate floral formation in maize and to be strongly associated with flowering time variation [44, 50, 51] (Additional file 2: Table S1).

### Candidate genes and GO enrichment analysis

**Fig. 3:**
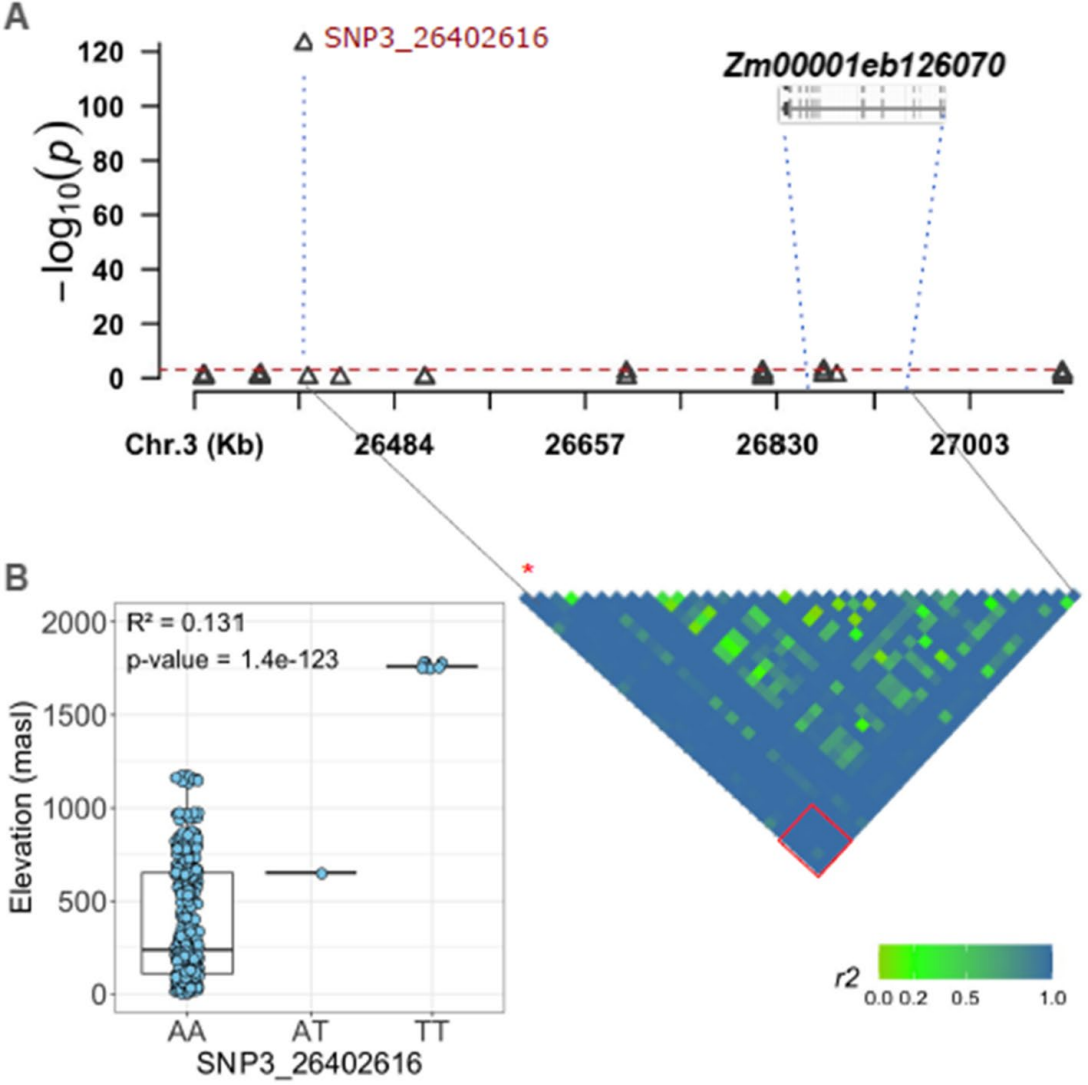
Local Manhattan plot of chromosome 3 (26,200,000–27,200,000 bp) highlighting the most significantly associated SNP, SNP3_26402616 (positioned at 26,402,616 bp), which shows a strong association with elevation and is strongly linked with the gene *Zm00001eb126070* (spanning 26,857,639 to 26,884,284 bp). Sub-figure A shows the linkage disequilibrium (LD) structure among 38 SNPs located between the significant SNP and the candidate gene. Sub-figure B illustrates that the T-allele of SNP3_26402616 is associated with adaptation to higher elevations, exhibiting an allelic effect of approximately 13%

Upon inspecting the topmost significant SNP for longitude on chromosome 5 at 171.3 Mb (*p*-value 1.32e − 84), we found a nearby gene model *Zm00001eb242340* (170.69–170.70 Mb) to be an ortholog of *AT1G12910* in *A. thaliana*, encoding for LIGHT-REGULATED WD1 (*LWD1*), a clock protein regulating the circadian period length and photoperiodic flowering. For elevation, the topmost SNP (*p*-value 1.4e − 123) on chromosome 3 at 26.4 Mb is strongly linked with *Zm00001eb126070* located (Fig. 3A). *Zm00001eb126070*, also an ortholog of *AT2G01130* in *A. thaliana*,is a member of the DEA(D/H)-box RNA helicase family protein expressed during petal differentiation. The minor allele of the SNP associated with *Zm00001eb126070* (26.85–26.88 Mb) was found to segregate within two southern French populations with high elevation (Fig. 3B).

**Fig. 4:**
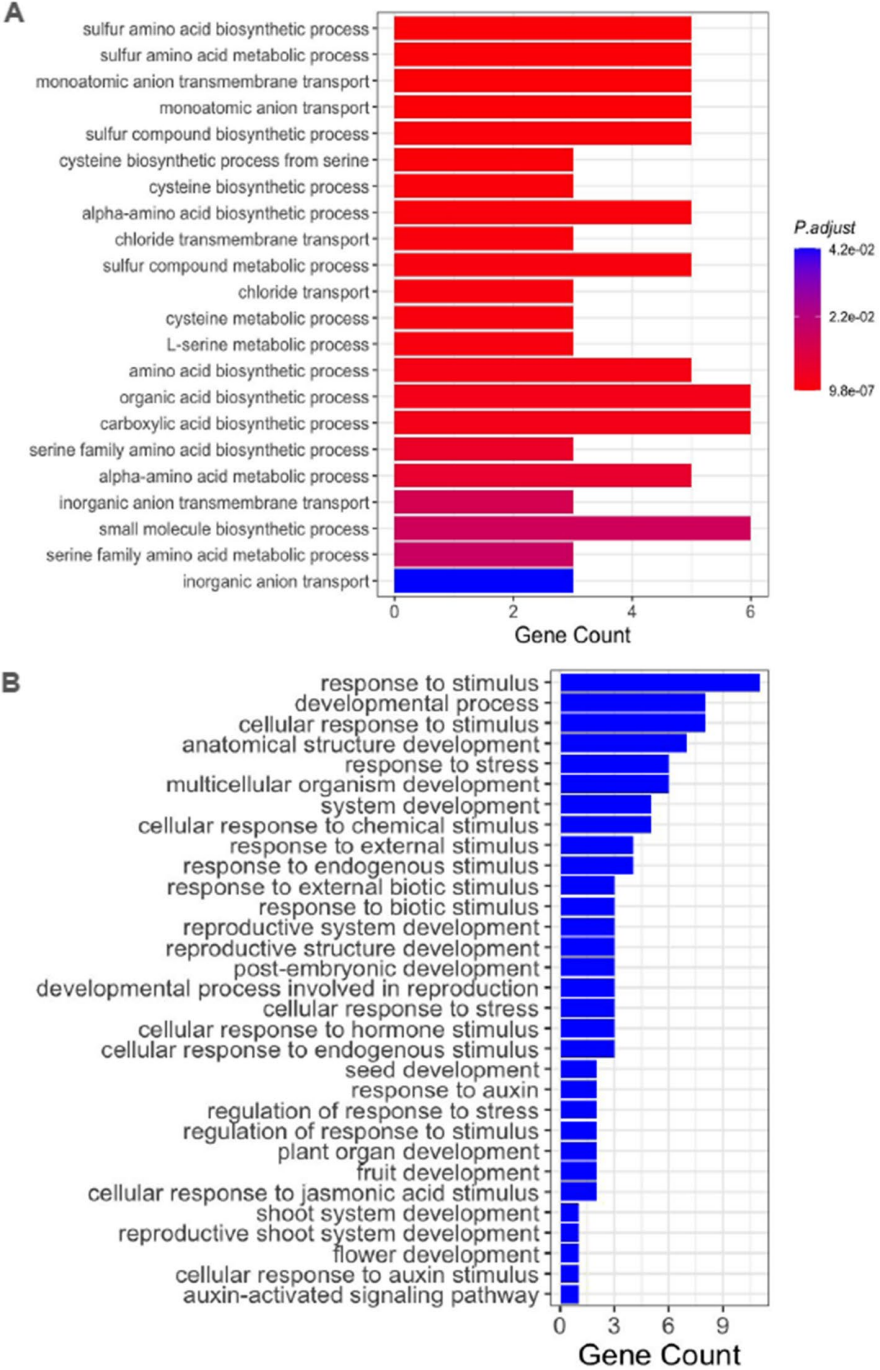
Enriched GO terms for 42 candidate genes associated with latitude, and significantly enriched at *p*-value < 0.05 (**A**). GO terms of some of the remaining candidate genes across latitude, longitude, and elevation that are not significantly enriched but indicative of local adaptation in maize (**B**)

Additionally, a search for candidate genes across all significantly associated SNPs using a window of ± 50 kb, led to the identification of 42, 39, and 45 associated candidate genes for latitude, longitude, and elevation, respectively. Leveraging the gene ontology (GO) files for B73 v5.0, we elucidated their biological functions, some of which describe local adaptation properties in maize. We conducted GO enrichment analysis to describe conserved GO terms of the identified genes. GO enrichment was successful for genes associated with latitude, as presented in Fig. 4A. However, elevation and longitude had no significantly enriched GO terms. The most enriched terms for latitude, namely"sulphur amino acid metabolic process"and"monoatomic anion transport,"play active roles in Anion channels/transporters, which are crucial to signaling pathways leading to the adaptation of plant cells to abiotic and biotic environmental stresses, as well as in the control of metabolism and maintenance of electrochemical gradients [52, 53].

## Discussion

### Geographic influence on genetic diversity of European maize landraces

Recent diversity studies on European maize have primarily focused on inbred lines derived from a limited number of landraces [18, 30, 34]. Earlier reports explored the genetic diversity of maize landraces in Europe, often from one or a few countries of origin or restricted geographic regions [18, 30, 34]. Some of these studies relied on marker types such as SSRs, RFLPs, and isozymes [9, 10, 12, 32, 42, 43, 54]. When the number of landraces was substantially large and originated from broader geographical regions, bulk genotyping of multiple individuals within a population was employed [17, 55], or SNP-array technology was used for genotyping single individuals [56]. Although significant efforts have been made to improve allele frequency estimation and ensure equal representation of individuals in bulk genotyping methods [16, 57], individual-level genetic variation remains crucial for understanding the genetic structure of traits within landrace populations and for identifying beneficial alleles with low frequencies. Additionally, the genotyping-by-sequencing (GBS) approach enables the discovery of novel and large-scale polymorphisms, which helps prevent misinterpretation of diversity studies due to ascertainment bias [19, 20].

In our study, we assessed the genetic structure of 40 European maize landrace populations (EMLPs) from nine countries of origin using 152,671 GBS SNP-markers from 333 genotyped individual plants. This provides a broad genetic base for evaluating EMLPs. The diversity in the geographic origins of these landraces, explaining 41% of the genetic variation as presented in the results, is comparable to 46% reported by Navarro et al. [5], although less than the 0.71 reported by Gauthier et al. [10], possibly due to differences in the type of genetic markers used. Nevertheless, these values underscore the significant impact of geographical spread on maize genetic diversity.

The use of genetic groups for the classification of EMLPs into northeastern, southeastern, south Italian, and the Pyrenees aligns with previous attempts at molecular classification of European maize [10, 16, 30, 44, 55]. In addition, we observed a mixture of the identified groups within countries and regions of origin. We anticipated that populations within a country would exhibit more similarities than populations from different countries; however, this was not the case. For instance, the mixture of two groups among the south Italian populations, attributed to their elevational differences, suggests that there exists remarkable genetic differentiation for adaptation to variable elevations of EMLPs. This, coupled with admixture observed in Bulgaria and Croatia, suggests that geographical proximity does not always guarantee genetic similarities. In other words, EMLPs expected to be on average more genetically similar might differ significantly in some divergent genomic regions which may contain genes enabling populations to adapt to slightly different environments. This highlights that selecting EMLPs for diversity representation is less effective when based solely on the country of origin but improves significantly when combined with variations in latitude, longitude, and elevation of origin. Conversely, group 2 comprised several closely related populations spanning four countries of origin (Additional file 1: Fig. S7). This mixture of countries within a group reveals the extent of serial exchange of materials and hybridization between tropical dents and temperate flints in the European corn-belt region [2, 30]. The insights gained from the clustering analysis should guide researchers and breeders in making informed selections of populations to study.

### Benefits of individual-plant-based genotyping for genetic studies

Due to the heterogeneity observed in maize landraces, as evident from the AMOVA result partitioning more than half of the genetic variance within the population, characterization should be carried out based on representative sets of individuals to efficiently capture the population’s diversity [5, 56–58]. This approach prevents the loss of information about individual plant genetic variation (Fig. 3B) and aids in identifying selection scans caused by linkage disequilibrium, which is limited in bulk genotyping [9, 59]. Gouda et al. [60] suggested a minimum of 5 individuals per population, and studies have used as low as 2 individuals [61], 6 individuals [62], and up to 30 individuals [42] for estimating maize population diversity. While cost considerations are relevant (which may become more affordable in the near future), genotyping individual plants is recommended over bulk genotyping for an optimal estimate of population diversity. Our EMLP individual plant panel allowed us to explore pairwise differences among individuals for estimating population diversity using *π* and assessing the degree of heterogeneity within populations. This measure of diversity has been reported as a reliable estimator, particularly when dealing with a large genome-wide dataset and a limited number of individuals per population [7, 44].

### Serial founder events during maize expansion in Europe

The gradual decline in π as latitude increases suggests a serial founder event as maize expanded from the sub-tropical south to the temperate north of Europe, possibly due to selection for adaptation to long days and lower temperatures [63]. Founder populations are a product of recurrent sub-sampling of diversity from preceding populations [64, 65]. A similar pattern was observed for American maize according to Wang et al. [33], where an extreme founder effect was observed for Andean populations based on their distance from the maize domestication center in southwestern Mexico. Serial founder events during maize expansion may have occurred when only a subset of whole ears, containing a fraction of the source population’s diversity, was carried to colonized regions [33, 66]. The movement of whole ears, composed of seeds that are full or maternal half-siblings, promotes “kin-structured” migration, increasing inbreeding by reducing the number of effective colonists [33, 67]. Gauthier et al. [10] reported two genomic regions driving European maize latitude variation using SSRs and a few other authors have documented the close population structure of Europe’s northern populations compared to the southern populations [9, 32, 43]. These studies further confirm that as maize migrated from centers of domestication across latitudinal gradients, genetic diversity progressively declined.

The report of a second introduction from North America [9, 12] aligns with the observation that the diversity of European maize represents ∼75% of that in the Americas. Several landraces cultivated in southwestern Europe are related to those of Mesoamerican origin, while landraces from northern Europe resemble North American flint varieties [2, 50]. Vigouroux et al. [61] found a similar reduction in genetic diversity as maize migrated from highland Mexico to the northern United States. This pattern of genetic diversity was also evident in the *F*_*ST*_ results, where the northeastern group showed the highest differentiation from other populations. Detailed *F*_*ST*_ results (Additional file 1: Fig. S8 and Additional file 1: Fig. S9) revealed that the farthest northeastern population from Hungary, with the lowest* π*, exhibited the greatest differentiation [68], corroborating the influence of serial founder effects. The maize migration patterns reported in this study provide a case study for understanding the adaptation of crops during global migration (e.g., out of the Americas) followed by continental migration (e.g., within Europe).

### Signatures of selection for local adaptation in European maize landraces

The most commonly used statistical methods for identifying selection signatures are *F*_*ST*_ outlier analysis and genetic-environment association analysis (GEA) [30, 69]. Here, we adopted the GEA approach, focusing on the association between SNPs and environmental variables under the assumption that genome-wide diversity primarily reflects the action of divergent selection, in this case, for local adaptation [5]. We employed a straightforward and rapid approach using the latitude, longitude, and elevation of the origin of geographically and genetically diverse populations to identify location-specific adaptation loci. This approach can be particularly useful in cases where budget is limited or logistical constraints exist.

The 443 significant associations identified were broadly distributed within and across chromosomes, affirming the polygenicity of local adaptation traits in maize [5]. Some of these loci aligned with reported loci for flowering time and plant height, as shown in Additional file 2: Table S1, supporting their strong relationship with maize adaptation [5, 7]. Agronomic traits, such as plant height and flowering time, have served as indicators of fitness for geographic and climatic variation [47, 49]. Janzen et al. [24] confirmed the relative home-site advantage displayed by local maize populations for various agronomic and vegetative traits, including plant height and flowering time, in a lowland versus highland site cross-reaction norm experiment. Hufford et al. [7] untangled the genomic targets for highland adaptation in maize on chromosome 4 (*In4vm*, between 150 and 200 Mb) as a result of gene introgression from the wild relative (*Zea Mexicana*). This introgression was later confirmed and shown to be significantly associated with flowering [5]. We found two significant associations within this 50 Mb region for elevation, suggesting a contribution to the adaptation of European maize to higher elevations.

Longitudinal variation of European maize accounted for majority of the associations, a discovery earlier reported by Gauthier et al. [10], who detected 5 SSR alleles for longitudinal differences compared to 2 SSR alleles for latitude, emphasizing the critical role of longitudinal distance in understanding European maize local adaptation.

During the domestication process, the *tb1* locus experienced a significant reduction in diversity, and the *Dwarf8* locus revealed signs of purifying selection accompanied by substantial diversity loss [70, 71]. While unverified, certain Northern Flint germplasms, such as sweet corn, exhibit a morphology resembling the undomesticated *tb1* phenotype. It is possible that the region encompassing *Dwarf8* and *tb1* underwent a bottleneck with multiple selective sweeps, leading to the formation of extended haplotype blocks for this region [72]. *ZCN7* and *ZCN8* are found in the photoperiod pathway and play a role in regulating flowering time [44, 73–75]. However, environmental variables such as precipitation, soil type, and soil microbes, among others, would have possibly introduced additional location-specific alleles associated with novel genes (e.g., *Zm00001eb242340 and Zm00001eb126070*) that were uncovered in the study.

## Conclusions

This study of European maize landrace populations (EMLPs) covering broad geographical regions provides a valuable resource for selecting representative populations that largely capture the gene pools of European maize diversity for breeding, conservation, and research. We conclude that latitude, elevation, and longitude are key factors in the genetic groupings of EMLPs, even within their country of origin. We propose genotyping multiple individuals per population for a thorough examination of the genetic parameters of the EMLP. We further revealed serial founding events that likely occurred during maize expansion in Europe due to selection. By using latitude, elevation, and longitude of origin as response variables, we identified both reported and novel SNP associations and genes potentially linked to local adaptation. Thus, in the absence of phenotypic information from field experiments with multi-environment replicates, this approach proved adequate for making informative associations for local adaptation traits. We have demonstrated the potential to use individual plants from maize landrace populations as a resource to gain insight into EMLPs’ genetic structure, selection events, and the genetic basis of their location-specific adaptation.

## Methods

### Plant material and experiment

Seeds from 40 European maize landrace populations (EMLPs) were collected from the Leibniz Institute of Plant Genetics and Crop Plant Research (IPK) germplasm repository (https://www.ipk-gatersleben.de/). These landraces were selected from nine countries of origin, namely Germany (5), France (3), Spain (2), Hungary (1), Croatia (8), Austria (1), Bulgaria (7), Turkey (1), and Italy (12) to cover broad geographical regions across latitudinal and longitudinal gradients of the continent (Additional file: Table S2). The experiment was laid out as an observation trial for phenotypic data by sowing 50 seeds per population in two rows on each plot at a distance of 15 cm by 95 cm. Based on the available number of germinated plants in each plot, a minimum of 3 to a maximum of 10 individual plants were sampled from each population 6 weeks after sowing, to be tracked through genotyping and phenotyping. Flowering time was measured as the number of days from sowing to visible shedding of pollen on tassel (days to anthesis) and to visible silk (days to silking). Plant height was scored at flowering as distance between the root crown and the node of the flag leaf. Distribution and Pearson’s correlation of traits are available in Additional file 1: Fig. S10. The experiment was conducted in Rosdorf, Göttingen, Germany (coordinates, 51.512679, 9.886327).

### Genotyping-by-sequencing

Leaf tissues were collected from a total of 340 individual plants across the 40 populations. These were collected from the youngest visible leaf 6 weeks after sowing and were lyophilized and submitted to the University of Wisconsin-Madison Biotechnology Center for DNA extraction using the Qiagen DNeasy 96 plant kit. DNA yield was quantified with Promega QuantiFluor on a Tecan Spark 10 M. DNA concentration was verified using the Quant-iT™ PicoGreen dsDNA kit (Life Technologies, Grand Island, NY). Libraries were prepared as in Elshire et al. [76] with minimal modification; in short, 150 ng of DNA was digested with *ApeK*I (New England Biolabs, Ipswich, MA) after which barcoded adapters amenable to Illumina sequencing were added by ligation with T4 ligase (New England Biolabs, Ipswich, MA). The 96 adapter-ligated DNA samples were pooled and amplified to provide library quantities amenable for sequencing, and adapter dimers were removed by SPRI bead purification. The quality and quantity of the finished libraries were assessed using the Agilent Bioanalyzer High Sensitivity Chip (Agilent Technologies, Inc., Santa Clara, CA) and Qubit dsDNA HS Assay Kit (Life Technologies, Grand Island, NY), respectively. Libraries were sequenced targeting approximately 300 million paired-end reads (per plate) on a Illumina HiSeq X, 2 X 150.

### Variant calling and filtering

Demultiplexing of the pooled raw reads dataset was done with sabre tools [77] using the barcode information. Demultiplexed reads were mapped to the B73 maize reference genome version 5.0 [78] using the Burrow Alignment tools (BWA-mem) [79]. Mapped reads were further trimmed and sorted using samtools [79]. Variants were called using bcftools [80]. Further filtering of the SNP data was performed using vcftools [80] to remove multi-allelic loci, and structural variants and retain only SNP with minor allele frequencies (MAF) greater than 0.01, QUAL scores > 30, minimum depth > 5, and maximum depth < 500. The average missingness proportion was 0.14. Imputation of missing values was done using Beagle v5.4 [81] and imputation accuracy was 0.96. SNP density across and within chromosomes was inspected using CMplot R -package [82]. Distribution of MAF and expected heterozygosity were performed using snpReady R -package [83].

### Genetic diversity and population structure

Genetic distance and geographic distance among the individual plants were estimated using individual plant SNP information and its population’s geographical data (latitude, longitude, and elevation of the collection site for each population), respectively. The Mantel test [39] was used to assess the relationship between the genetic and geographic distances of the populations. Hierarchical clustering analysis was performed using the genetic distance matrix for population structure analysis, as implemented in the R package ‘ape’ v5.0 [84]. Multidimensional scaling analysis was conducted using principal coordinates to inspect the clustering of individuals and populations [85].

Country of origin, year of collection, collection site, latitude, longitude, and elevation of the collection sites for each population, as provided in the passport data (Additional file: Table S2), were further examined to explain the population structure. Analysis of molecular variance (AMOVA) was used to investigate the partitioning of the genetic variation within and between populations and groups, using the poppr R package v2.9.4 [86]. Fixation indices (F_ST_) among groups (each consisting of two or more populations) and among populations (each consisting of 3–10 individual plants) were estimated using the Hierfstat R package v0.5–11 [87]. For the population’s genetic diversity, nucleotide diversity (π), the average pairwise genetic differences (using Rogers distance) [88] between individual plants within a population were used. Pearson’s correlation was applied to assess the relationship between π and latitude, longitude, elevation of origin, and traits such as days to anthesis, days to silking, and plant height of the populations (Additional file 1: Fig. S10).

### GWAS, selection signature, and search for candidate genes

Genome-wide association analysis was conducted by adopting the latitude, elevation, and longitude of origin of the populations as response variables (assuming genetic adaptation of the population to elevation, latitude, and longitude of the collection sites as the traits) and the SNP markers of the individual plants as explanatory variables. Fixed and Random Model Circulating Probability Unification (FarmCPU), implemented in the GAPIT3 R-package [46], was used to scan the SNP markers for association using the first three principal coordinates as covariates. FarmCPU performs a single marker scan with associated markers as cofactors in a fixed effect model and independently optimizes the associated cofactors in a random effect model. This helps to correct for multiple testing errors and reduce the risk of compromising the true positives as is the case in mixed-linear models (MLM) [46]. Significant SNPs were defined based on a significance threshold of *P* < 0.00001. This means that we expect fewer than three false positives based on the size of our SNP dataset. A window of ± 50 kb was selected, analogous with the threshold used in a previous study [5], to identify genes potentially linked to significant SNPs. This was further validated by analyzing the LD (using r2) between all pairs of SNPs within the ± 50 kb window for one of the significant SNPs. Genes found within this window were functionally annotated for ontology terms using the gostprofiler R-package [89] using the gff3 file from Ensembl (https://ensembl.gramene.org/Zea_mays/Info/Index) and the maize genome database (https://www.maizegdb.org/genome/assembly/Zm-B73-REFERENCE-NAM-5.0).

## Supplementary Information

Additional file 1: Figure S1-S10. Figure S1. Diversity in seed color, size, and shape of the 40 landrace populations used for this studyand the percentage representation of each country in the study panel. Figure S2. Distribution of minor allele frequenciesand expected heterozygosityof the 152,671 SNP markers. Figure S3. Line graph showing K= 5 has the optimal number of genetic groups using the K-means clustering algorithm. Figure S4. Distribution of latitude, elevation, and longitude across the five genetic groups. Figure S5. Analysis of molecular variance showing the partitioning of genetic variance among groups, among populations within groups, and among individuals within populations. Figure S6. EMLP’s nucleotide diversity, colored by genetic groups, illustrates the degree of genetic heterogeneity within each population. The number of individual plants in each population is displayed above the whisker boxes. Black dot within boxes is the mean π of each population. Figure S7. Principal coordinate analysis showing mixtures of genetic groups within countries of origin. Figure S8. FST showing the genetic differentiation between the 40 EMLPs. Figure S9. FST showing the genetic differentiation between the nine countries of origins of EMLPs. Figure S10. Correlation among three traits days to anthesis, days to silking, plant height, and nucleotide diversity, and the latitude, longitude, and elevationof origin of the 40 populations.

Additional file 2: Table S1-S2. Table S1. List of reported QTLs and genes for flowering time and plant height that overlaps with the significantly associated SNPs found in this study. Table S2. Passport data of the 40 European maize landrace populations used for this study [90–94].

## Data Availability

GBS sequence data for each individual is available at: https://www.ncbi.nlm.nih.gov/sra/?term=PRJNA1224704. Final SNP dataset and passport information are available at: https://figshare.com/account/home#/collections/7803434. Code for main analysis are available at: https://github.com/Aiyesa/EMLP-local-adaptation.

## Abbreviations

AMOVA: Analysis of molecular variance
EMLP: European Maize Landrace Population
FarmCPU: Fixed and Random Model Circulating Probability Unification
FST: Fixation index
GBS: Genotyping-by-sequencing
GEA: Genotype environment association
GO: Gene ontology
GWAS: Genome-wide association studies
IP: Individual plant
LD: Linkage disequilibrium
MADS69: MADS-box transcription factor 69
MAF: Minor allele frequency
PCoA: Principal coordinate analysis
SNP: Single Nucleotide Polymorphism
SSR: Single sequence repeat
tb1: teosinte branched 1
ZCN7: ZEA CENTRORADIALIS7
ZCN8: ZEA CENTRORADIALIS8
