# Supplementary material for: Individual plant genetics reveal the control of local adaptation in European maize landraces
Source: BMC Biol. 2025 May 21;23:138. doi: 10.1186/s12915-025-02241-8 (PMC12096487; doi:10.1186/s12915-025-02241-8)
Supplement: Supplementary file 1 — Additional file 1: Figure S1-S10. Figure S1. Diversity in seed color, size, and shape of the 40 landrace populations used for this studyand the percentage representation of each country in the study panel. Figure S2. Distribution of minor allele frequenciesand expected heterozygosityof the 152,671 SNP markers. Figure S3. Line graph showing K= 5 has the optimal number of genetic groups using the K-means clustering algorithm. Figure S4. Distribution of latitude, elevation, and longitude across the five genetic groups. Figure S5. Analysis of molecular variance showing the partitioning of genetic variance among groups, among populations within groups, and among individuals within populations. Figure S6. EMLP’s nucleotide diversity, colored by genetic groups, illustrates the degree of genetic heterogeneity within each population. The number of individual plants in each population is displayed above the whisker boxes. Black dot within boxes is the mean π of each population. Figure S7. Principal coordinate analysis showing mixtures of genetic groups within countries of origin. Figure S8. FST showing the genetic differentiation between the 40 EMLPs. Figure S9. FST showing the genetic differentiation between the nine countries of origins of EMLPs. Figure S10. Correlation among three traits days to anthesis, days to silking, plant height, and nucleotide diversity, and the latitude, longitude, and elevationof origin of the 40 populations. [file 12915_2025_2241_MOESM1_ESM.docx]

# Supplementary Tables

Additional file: Table S1. List of reported QTLs and genes for flowering time and plant height that overlaps with the significantly associated SNPs found in this study.

| Geographical feature | Significant SNPs | Chromosome | Number of significant SNPs | SNP position | Pvalue | Reported QTL | Reported position (Mb) | Reported trait | Reference |
| --- | --- | --- | --- | --- | --- | --- | --- | --- | --- |
| Latitude | SNP1_224875774 | 1 | 1 | 224875774 | 1.20E-22 | Dwarf8 | 221 - 227.0 | Plant height | [92] |
| Longitude | SNP1_221583765,  SNP1_224956653,  SNP1_224956770,  SNP1_225739065 | 1 | 4 | 221583765,  224956653,  224956770,  225739065 | 3.34E-06,  4.94E-06,  1.83E-07,  2.05E-06 | Dwarf8 | 221 - 227.0 | Plant height | [93] |
|  |  |  |  |  |  |  |  |  |  |
| Longitude | SNP3_162270806 | 3 | 1 | 162270806 | 2.40E-09 | ZmMADS69 | 161.2 | Flowering time | [94] |
|  |  |  |  |  |  |  |  |  |  |
|  |  |  |  |  |  |  |  |  |  |
| Elevation | SNP4_162880690,  SNP4_163298632 | 4 | 2 | 162880690,  163298632 | 5.93E-06,  2.91E-06 | Inv4m | 160 - 190 | Flowering time | [5,51] |
| Longitude | SNP4_169160985,  SNP4_189097788 | 4 | 2 | 169160985,  189097788 | 4.93E-07,  6.75E-06 | Inv4m | 160 - 190 | Flowering time | [5,51] |
|  |  |  |  |  |  |  |  |  |  |
| Longitude | SNP1_262301372,  SNP1_263734924,  SNP1_264903088,  SNP1_264903089 | 1 | 4 | 262301372,  263734924,  264903088,  264903089 | 8.49E-06,  8.98E-16,  1.39E-06,  2.15E-06 | tb1 | 265.8 | plant elongation | [52] |
| Elevation | SNP1_260934042,  SNP1_267878222 | 1 | 2 | 260934042,  267878222 | 8.76E-06,  7.90E-06 | tb1 | 265.8 | plant elongation | [52] |
|  |  |  |  |  |  |  |  |  |  |
| Longitude | SNP5_171332533 | 5 | 1 | 171332533 | 1.32E-84 | qHT_YZ5a | 147.4–217.3 | Plant height | [92] |
| Latitude | SNP5_163900212,  SNP5_195957630 | 5 | 2 | 163900212,  195957630 | 9.83E-06,  6.91E-08 | qHT_YZ5a |  | Plant height | [92] |
|  |  |  |  |  |  |  |  |  |  |
| Latitude | SNP6_171329622 | 6 | 1 | 171329622 | 2.50E-06 | ZCN7 | 170.7 | Flowering time | [95] |
|  |  |  |  |  |  |  |  |  |  |
| Longitude | SNP10_78115698 | 10 | 1 | 78.1 | 3.20E-06 | ZmCCA1a | 78.3 | Flowering time | [75] |
|  |  |  |  |  |  |  |  |  |  |
| Longitude | SNP8_128506452 | 8 | 1 | 129 | 1.81E-08 | ZCN8 | 120 - 150 | Flowering time | [76] |
|  |  |  |  |  |  |  |  |  |  |
| Latitude | SNP7_34066038 | 7 | 1 | 34066038 | 1.50E-10 | QTN | 34.5 | Plant height | [96] |
|  |  |  |  |  |  |  |  |  |  |
| Latitude | SNP7_156505045,  SNP7_156505965,  SNP7_160221995 | 7 | 3 | 156505045,  156505965,  160221995 | 5.91E-06,  5.06E-06,  6.36E-09 | hDS7 | 152.1 - 159.9 | Days to silking | [96] |
|  |  |  |  |  |  |  |  |  |  |
| Elevation | SNP4_223453624,  SNP4_223453628,  SNP4_223453774 | 4 | 3 | 223453624,  223453628,  223453774 | 5.67E-06,  7.02E-06,  1.80E-06 | QTN | 223 | Plant height | [97] |
