# Supplementary material for: Individual plant genetics reveal the control of local adaptation in European maize landraces
Source: BMC Biol. 2025 May 21;23:138. doi: 10.1186/s12915-025-02241-8 (PMC12096487; doi:10.1186/s12915-025-02241-8)
Supplement: Supplementary file 2 — Additional file 2: Table S1-S2. Table S1. List of reported QTLs and genes for flowering time and plant height that overlaps with the significantly associated SNPs found in this study. Table S2. Passport data of the 40 European maize landrace populations used for this study [90–94]. [file 12915_2025_2241_MOESM2_ESM.docx]

Additional file: Table S2. Passport data of the 40 European maize landrace populations used for this study.

| **Populations** | **Accession no** | **Country** | **Collection site** | **Collection date** | **Common name** | **Latitude (^o^N)** | **Longitude (^o^E)** | **Elevation (masl)** |
| --- | --- | --- | --- | --- | --- | --- | --- | --- |
| GER_003 | ZEA 3 | Germany | Nossen | 1956 | Gelber-badischer | 51.06 | 13.29 | 250 |
| GER_065 | ZEA 65 | Germany | Landau-der-Pflaz | 1950 | NA | 49.29 | 7.89 | 263 |
| ITA_1027 | ZEA 1027 | Italy | Cosenza | 1985 | quarantina | 39.73 | 16.17 | 238 |
| ITA_1033 | ZEA 1033 | Italy | San-giovanni-fiore | 1985 | Miglo | 39.25 | 16.71 | 950 |
| ITA_1049 | ZEA 1049 | Italy | Catanzaro | 1985 | granoturco | 38.12 | 15.64 | 350 |
| ITA_1050 | ZEA 1050 | Italy | Santa-cristina-dapromonte | 1985 | radindia† | 38.25 | 16.96 | 700 |
| ITA_1051 | ZEA 1051 | Italy | Bagaladi | 1985 | Panico | 38.02 | 15.76 | 1150 |
| ITA_1086 | ZEA 1086 | Italy | Castiglione-agnone | 1988 | nostrale | 42.02 | 14.4 | 800 |
| AUT_1122 | ZEA 1122 | Austria | Salzburg | 1990 | NA | 47.83 | 13.05 | 424 |
| ITA_1149 | ZEA 1149 | Italy | Roncaglia | 1991 | pignoletto | 45.01 | 9.78 | 484 |
| HUN_116 | ZEA 116 | Hungary | Debrecen | 1952 | Black-sugar | 47.63 | 21.65 | 121 |
| ITA_3302 | ZEA 3302 | Italy | Barile | 1996 | gradino | 40.95 | 15.69 | 600 |
| ITA_3307 | ZEA 3307 | Italy | S-Costantino-albanese | 1996 | cuccian | 40.02 | 16.26 | 650 |
| HRV_3351 | ZEA 3351 | Croatia | Drenovci | 1996 | bjeli | 44.92 | 18.91 | 100 |
| BGR_3368 | ZEA 3368 | Bulgaria | Simitli | 1998 | Carevitsa | 41.53 | 23 | 290 |
| BGR_3373 | ZEA 3373 | Bulgaria | Haskovo | 1998 | Bodliva | 42.02 | 25.41 | 60 |
| BGR_3376 | ZEA 3376 | Bulgaria | Dimitrovgrad-haskovo | 1998 | mesna | 42.29 | 25.53 | 60 |
| BGR_3377 | ZEA 3377 | Bulgaria | Jasna-polyjanas | 1998 | mamuli | 42.25 | 27.57 | 20 |
| BGR_3378 | ZEA 3378 | Bulgaria | Josna-poljana | 1998 | mamuli | 42.17 | 27.3 | 20 |
| BGR_3387 | ZEA 3387 | Bulgaria | Nordbulgarien | 1998 | Konski-sab | 43.32 | 25.52 | 200 |
| BGR_3390 | ZEA 3390 | Bulgaria | Sadovo-Plovdiv | 1998 | pakliwa-zarewiza | 42.14 | 24.93 | 30 |
| ITA_3430 | ZEA 3430 | Italy | Marsico-Nuovo | 1992 | popcorn | 40.56 | 15.65 | 750 |
| ESP_3439 | ZEA 3439 | Spain | Galizien | 1999 | Blanco | 42.94 | -8.59 | 560 |
| ESP_3538 | ZEA 3538 | Spain | CITA-Valle-Guerra-Teneriffa | 1994 | Mais-von-Lanzarote | 28.98 | -16.46 | 218 |
| HRV_3556 | ZEA 3556 | Croatia | Drenovci | 1996 | crverni | 44.92 | 18.92 | 100 |
| HRV_3560 | ZEA 3560 | Croatia | Komeletinci | 1996 | kokicar | 45.3 | 18.81 | 200 |
| HRV_3561 | ZEA 3561 | Croatia | Gorjani | 1996 | za-kuhanje | 45.31 | 18.44 | 220 |
| HRV_3564 | ZEA 3564 | Croatia | CigocHˆhe | 1996 | bijeli-domaci-kukuruz | 45.43 | 16.63 | 80 |
| HRV_3565 | ZEA 3565 | Croatia | CigocHˆhe | 1996 | Crveni | 45.42 | 16.63 | 80 |
| HRV_3567 | ZEA 3567 | Croatia | Petrusevec | 1996 | kukuruz-bijeli | 45.78 | 16.05 | 220 |
| HRV_3568 | ZEA 3568 | Croatia | Petrusevec | 1996 | kukuruz-zuti | 45.78 | 16.06 | 180 |
| ITA_3573 | ZEA 3573 | Italy | Ponza-Contrada-Conti | 1999 | grantin | 40.9 | 12.96 | 538 |
| TUR_3602 | ZEA 3602 | Turkey | Sapanca-Feld | 2003 | NA | 40.68 | 30.24 | 56.33 |
| GER_373 | ZEA 373 | Germany | East-Germany | 1975 | NA | 52.4 | 12.5 | 110 |
| FRA_688 | ZEA 688 | France | Bareilles | 1973 | NA | 42.9 | 0.42 | 1760 |
| FRA_693 | ZEA 693 | France | Massat | 1973 | NA | 42.88 | 1.43 | 651 |
| FRA_695 | ZEA 695 | France | Viey | 1973 | NA | 42.88 | 0.24 | 787 |
| GER_707 | ZEA 707 | Germany | East-Germany | 1975 | NA | 52.4 | 12.5 | 110 |
| GER_730 | ZEA 730 | Germany | East-Germany | 1975 | NA | 52.4 | 12.5 | 110 |
| ITA_786 | ZEA 786 | Italy | Salerno | 1983 | Quarantina | 40.22 | 15.36 | 850 |
